# Determination of the Optimal Sensing Temperature in Pt/Ta_2_O_5_/MoO_3_ Schottky Contacted Nanobelt Straddling Heterojunction

**DOI:** 10.3390/s18113770

**Published:** 2018-11-05

**Authors:** Ka Wai Cheung, Jerry Yu, Derek Ho

**Affiliations:** Department of Materials Science and Engineering, City University of Hong Kong, 88 Tat Chee Avenue, Kowloon Tong, Kowloon, Hong Kong; kwcheun44-c@my.cityu.edu.hk (K.W.C.); jcwyu@ieee.org (J.Y.)

**Keywords:** nanobelt, heterojunction, Schottky barrier, Ta_2_O_5_, MoO_3_, optimal sensing temperature

## Abstract

Nanostructured Schottky barrier gas sensors have emerged as novel semiconductor devices with large surface areas and unique electronic characteristics. Although it is widely known that operating these gas sensors requires heating to an optimal temperature for the highest sensitivity, the fundamental mechanism that governs the temperature-dependent sensitivity has yet been well understood. In this work, we present new evidence to support that thermionic field emission (TFE) is the dominant transport mechanism for Schottky contacted nanostructured heterojunction gas sensors at their optimal sensing temperature. Through the fabrication and characterization of Pt/MoO_3_ Schottky contacts, and Pt/Ta_2_O_5_/MoO_3_ heterojunctions, we found a previously unreported connection between TFE transport and optimal gas sensing temperature. This connection enables the description of Schottky barrier gas sensing performance using transport theory, which is a major step towards systematic engineering of gas sensors with nanostructured high-*k* oxide layers.

## 1. Introduction

The semiconductor gas sensor is an important class of devices, with applications in the environmental, energy, industrial, and medical industries [1,2]. In these sensors, multi-layered, low-dimensional, and nanostructured thin-films of metal oxides have been shown to achieve new capabilities and higher performance compared to their bulk counterparts [3]. The surface morphology and engineered defects of these oxides provide crucial increases in the surface area for interaction with target gas molecules. As these devices scale down to smaller dimensions, the advantage of a large surface area is especially pronounced. The unique properties of nanostructured oxides allow them to excel in terms of sensitivity, size, and power consumption [4,5]. In addition, these devices can be made compatible with existing silicon microelectronic technologies, enabling potential monolithic integration with readout circuits.

Schottky barrier sensors can be developed by depositing large work function noble metals, such as platinum (Pt), gold (Au) and palladium (Pd), as electrodes [6,7,8]. The operating principles of the Schottky barrier sensors are primarily associated with the observation of the shifts in the current-voltage (I–V) curve. Specifically, the dissociation of the gas analyte on the catalytic metal surface alters the Schottky barrier height at the metal/metal-oxide interface [3,9], which results in a current change across the interface.

The gas sensing characteristics of the Schottky barrier sensors are greatly influenced by the surface morphology of the interface [10,11]. Nanostructured Schottky barriers exhibit significantly higher sensitivity than their thin-film counterparts. For example, Ma et al. recently reported a ZnO/In_2_O_3_ heterojunction based methanol sensor [12]. Nanostructured forms of flower-like CuO/ZnO nanorods were reported by Zhang et al. [13] for ethanol sensing. Thus far, there are only limited reports on heterojunction sensors for nitrogen dioxide [14], triethylamine [15] and hydrogen [16,17].

High-*k* materials, such as tantalum oxide (Ta_2_O_5_), can be used to enhance sensitivity. Specifically, high-*k* materials can be engineered to have a large number of defects, which provide energy states in the middle of the bandgap [18,19,20,21]. Taking hydrogen sensing as an example, upon the adsorption of H^+^ ions, the generated electrons can undergo trapping and detrapping as they transport through the defect-rich interface [22]. The augmentation of transport improves sensitivity, as experimentally shown in an La_2_O_3_/SnO_2_ structure for CO_2_ sensing [23]. However, due to the change in transport mechanism, analysis using field emission (FE) or thermionic emission (TE) theory, as it is conventionally done, is therefore no longer adequate. For example, the brute force uses of FE or TE to describe these structures may lead to nonsensical parameter values. Recently, Poole-Frenkel (PF) theory has been proposed for characterizing nanostructured molybdenum oxide (MoO_3_) nanoplatelet sensors [24], and W doped Nb_2_O_5_ nanorods [25,26]. However, the extracted dielectric constants are inconsistent with well-established references, revealing the limitations of PF theory. Although the literature has shown many effective materials and techniques for metal-oxide sensors, the fundamental mechanism that governs the temperature-dependent sensitivity of Schottky contacted metal-oxide heterojunctions has yet been well understood.

In this work, we propose thermionic field emission as the dominant transport mechanism for Schottky contacted nanostructured heterojunction gas sensors at their optimal sensing temperature. Through the fabrication and characterization of a Pt/MoO_3_ Schottky contacts and a Pt/Ta_2_O_5_/MoO_3_ heterojunctions, we found a previously unreported connection between thermionic field emission (TFE) transport and optimal gas sensing temperature. This connection enables the optimization of Schottky barriers gas sensing performance using classical carrier transport theory, which is a powerful analytical tool for the systematic engineering of semiconductor gas sensors.

## 2. Materials and Methods

The Pt/Ta_2_O_5_/MoO_3_ nanostructured Schottky diode bulk device structures were fabricated via several steps, in order: substrate preparation, nanostructure synthesis, Ta_2_O_5_ layer deposition, and metal contact formation. As an initial fabrication step, an *n*-type <100> Silicon (Si) wafers (University Wafer) of 0.001–0.005 Ω·cm were cleaned with 10% hydrofluoric (HF) acid in water (H_2_O), to remove the native silicon dioxide (SiO_2_) layer. To form ohmic contacts, titanium (Ti) and platinum (Pt) layers were first deposited by radio frequency (RF) sputtering, then annealed at 500 °C in air for 30 min. The resulting ohmic contact consists of Ti/Pt (40 nm/100 nm). The wafers were diced into 10 mm × 10 mm substrates using a Disco DAD-321 dicing saw, and the polished front side of the Si was cleaned with 40% HF in H_2_O to remove any surface artifacts from the substrates.

The sensing layers were then deposited. Deposition of MoO_3_ nanobelts was performed by thermal evaporation (Lenton). MoO_3_ powder (Zheng Zhou) was placed inside a quartz tube and the Si substrate was placed 14 cm from the source. Deposition was performed by heating the tube furnace to 775 °C for 30 min at a flow rate of 0.7 L/min in a 10% O_2_/90% Ar gas mixture (Linde). To improve gas sensing, a Ta_2_O_5_ layer was deposited onto the MoO_3_ nanobelts by RF sputtering (Denton Discovery), under a low vacuum of 5 ×10^−7^ Torr and using an RF power of 20 W. A crystal thickness monitor was utilized to control the deposition thickness to 4 nm. Pt contact was sputtered via a shadow mask (1 mm diameter) into a thickness of 30 nm, then annealed at 300 °C. The dimensions of the fabricated structure were Pt/Ta_2_O_5_/MoO_3_-nanobelts (30 nm/4 nm/20 μm) on Si (250 μm). 

The surface morphology, Ta_2_O_5_ layer thickness, crystallographic structure and stoichiometric composition were characterized by scanning electron microscopy (SEM), transmission electron microscopy (TEM), X-ray diffraction (XRD) and X-ray photoelectron spectroscopy (XPS), respectively. I–V measurements were performed using a semiconductor parameter analyzer (Keithley 4200 series).

Gas testing of the Pt/MoO_3_ and Pt/Ta_2_O_5_/MoO_3_ devices was as follows. Prior to testing, dry (zero humidity) synthetic air (Scientific Gas Engineering Co.) was allowed to flow into a custom-built gas sealed stainless steel test chamber containing the devices with a total volume of 200 mm^3^. The maximum flow rate was maintained at 0.2 L/min using a mass flow controller (MKS Instruments). A conductive ceramic planar heater was placed under the devices to control the temperature in the range of 25 to 300 °C. Gas calibration tests was performed by diluting the flow of H_2_ gas with synthetic air to concentrations of 625, 1200, 2500, 5000 and 10,000 ppm. 

## 3. Results

**Figure 1:**
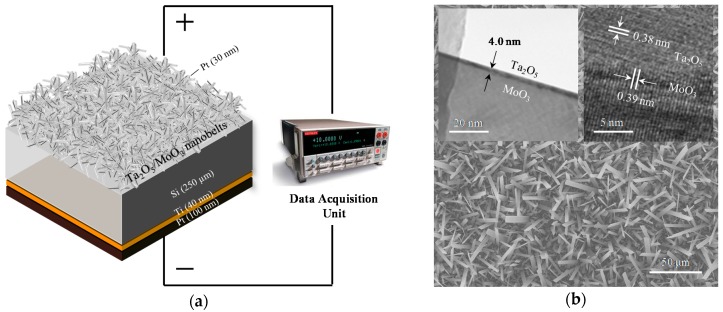
(**a**) Device structure with connection to the data acquisition unit. For the Schottky contact, Pt is deposited directly on the MoO_3_ nanobelts. For the heterojunction, Ta_2_O_5_, then Pt, are deposited. (**b**) Scanning electron microscopy (SEM) of the MoO_3_ nanobelts depicting average lengths and widths of 20 µm and 2 µm, respectively. The left inset shows a transmission electron microscopy (TEM) of the high-*k* layer with a thickness of 4 nm. The right inset shows the high resolution TEM (HRTEM) of the Ta_2_O_5_/MoO_3_ nanobelt interface with inter-planar atomic distances for these two materials at 0.38 nm and 0.39 nm, respectively.

Figure 1a shows the structure of the device and its connection to a source meter for acquisition of I–V data. For the Schottky contact reference device, the Pt layer is directly on the MoO_3_ nanobelts. For the heterojunction, a Ta_2_O_5_ layer is sandwiched between Pt and MoO_3_ nanobelts. Figure 1b shows the SEM of the MoO_3_ nanobelts depicting average lengths and widths of 20 µm and 2 µm, respectively. The left inset shows a TEM image of the high-*k* layer with a thickness of 4 nm. The right inset shows an image from high resolution TEM (HR-TEM) of the Ta_2_O_5_/MoO_3_ nanobelt interface, with inter-planar atomic distances for these two materials being 0.38 nm and 0.39 nm, respectively. Morphological and structural characterizations show that the controlled growth of a thin layer of Ta_2_O_5_ on the MoO_3_ nanobelts was achieved with highly ordered atomic arrangement.

**Figure 2:**
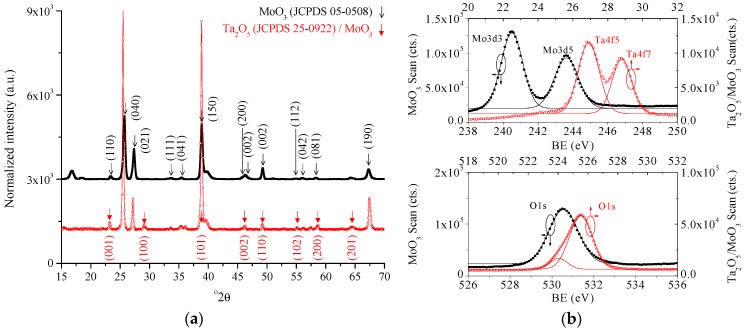
(**a**) X-ray diffraction (XRD) of the MoO_3_ nanobelts and Ta_2_O_5_/MoO_3_ nanobelts. (**b**) X-ray photoelectron spectroscopy (XPS) of the Ta4f5 and Ta4f7 binding energy peaks from the Ta_2_O_5_/MoO_3_ nanobelts, and the XPS of the Mo3d3 and Mo3d5 binding energy peaks from the MoO_3_ nanobelts (upper figure). XPS of the O1s binding energy peaks of MoO_3_ nanobelts and Ta_2_O_5_/MoO_3_ nanobelts (lower figure).

Crystallographic structure is then investigated. The XRD diffractogram of the MoO_3_ nanobelts and Ta_2_O_5_/MoO_3_ nanobelts are presented in Figure 2a. The crystallography of the MoO_3_ nanobelts can be correlated to the orthorhombic structure JCPDS#05-0508 and the arrows in the diffractogram show the presence of a Ta_2_O_5_ layer on the nanobelts. The top part of Figure 2b shows the XPS of the Ta4f5 and Ta4f7 binding energy peaks from the Ta_2_O_5_/MoO_3_ nanobelts, and the XPS of the Mo3d3 and Mo3d5 binding energy peaks from the MoO_3_ nanobelts. The bottom part of Figure 2b shows the XPS of the O1s binding energy peaks of MoO_3_ nanobelts and Ta_2_O_5_/MoO_3_ nanobelts. The binding energy data, fitted using a Gaussian model, indicates the presence of stoichiometric MoO_3_ (evident from the symmetry in the O1s peak) and substoichiometric Ta_2_O_5_ (evident from the shoulder peak at 530.3 eV). Results indicate the presence of stoichiometric polycrystalline MoO_3_ and a Ta_2_O_5_ layer consisting of defects due to its substoichiometric composition.

**Figure 3:**
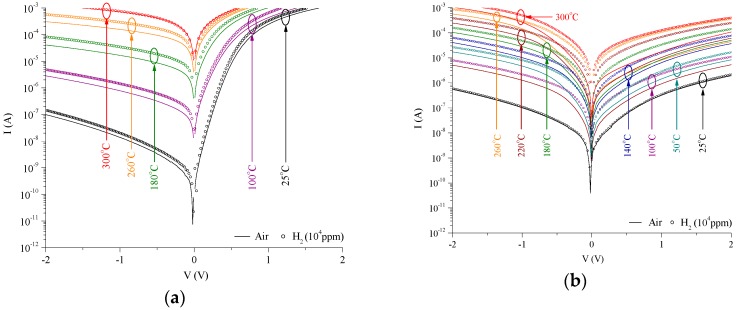
Current-Voltage (I–V) characteristics of the (**a**) Pt/MoO_3_ Schottky contact, and (**b**) Pt/Ta_2_O_5_/MoO_3_ heterojunction towards air and H_2_ (10,000 ppm) across temperature.

Static gas response of the devices across temperature is presented in Figure 3. As the sensor is exposed to hydrogen, the H_2_ molecules are thermodynamically adsorbed and catalytic dissociated at the Pt surface. Subsequent diffusion and adsorption of hydrogen ions (H^+^) at the Pt/MoO_3_ or Pt/Ta_2_O_5_ interfaces result in the migration of charge from the H^+^ donors to the oxygen (O^2−^) species, effectively creating dipolar charges at the interface. These dipolar charges lower the Schottky barrier, giving rise to a measurable potential difference (ΔV). Figure 3a,b shows the I–V characteristics across 25–300 °C in air and 10,000 ppm of H_2_ for the Pt/MoO_3_ Schottky contact and Pt/Ta_2_O_5_/MoO_3_ heterojunction, respectively. The Schottky diode shows strong rectifying characteristics in forward bias, whereas the heterojunction appears to exhibit stronger rectifying characteristics in reverse bias. In Figure 3b, the reverse current is greater than the forward current, which can be attributed to a scale down effect of nanostructured interfaces as explored previously [27]. The largest shift in the I–V characteristics are at 180 °C for the Pt /MoO_3_ device and 260 °C for the Pt/Ta_2_O_5_/MoO_3_ device. These are defined as the optimal operating temperatures.

**Figure 4:**
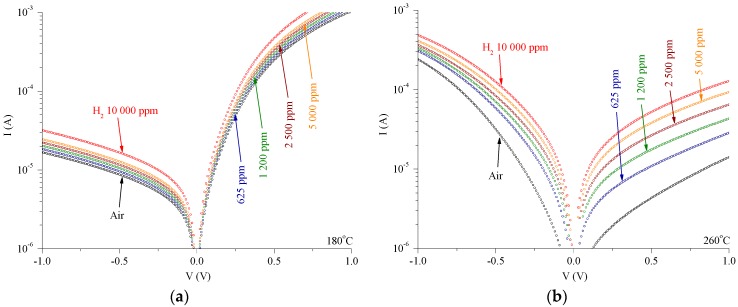
I–V characteristics in air and H_2_ for (**a**) the Pt/MoO_3_ Schottky contact and (**b**) the Pt/Ta_2_O_5_/MoO_3_ heterojunction, at their respective optimal operating temperatures of 180 °C and 260 °C. The H_2_ gas concentrations are 625, 1200, 2500, 5000 and 10,000 ppm.

**Figure 5:**
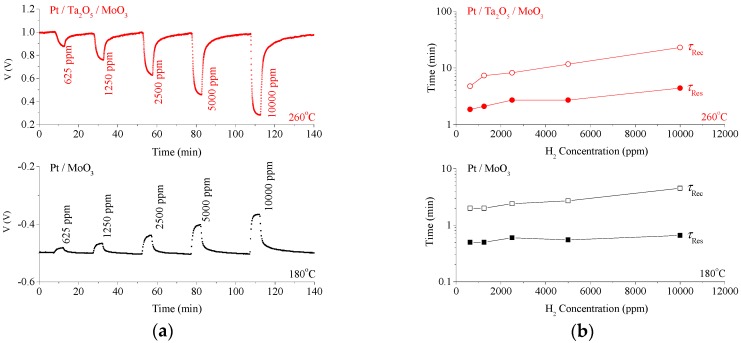
(**a**) Forward bias (+10 μA) dynamic H_2_ gas response of the (**a**) Pt/Ta_2_O_5_/MoO_3_ heterojunction diode at 260 °C, to concentrations of 625, 1250, 2500, 5000 and 10,000 ppm (upper figure). Response time τ_Res_ and recovery time τ_Rec_ of the heterojunction across H_2_ concentrations (lower figure). (**b**) Reverse bias (–10 μA) dynamic response towards H_2_ gas of the Pt/MoO_3_ Schottky contact at 180 °C under the aforementioned concentrations (upper figure). τ_Res_ and τ_Rec_ of the Schottky contact across H_2_ concentrations (lower figure).

I–V characteristics across H_2_ concentrations for the Pt/MoO_3_ Schottky contact and the Pt/Ta_2_O_5_/MoO_3_ heterojunction are presented in Figure 4a,b, respectively. Measurements were obtained at the optimal operating temperature. Figure 5a,b depicts the dynamic gas response of the heterojunction and Schottky contact, under 10 μA (forward) and −10 μA (reverse) biases, respectively. Figure 5a shows the voltage response with respect to H_2_ gas under the concentrations of 625, 1200, 2500, 5000 and 10,000 ppm. Figure 5b shows the response time τ_Res_ and recovery time τ_Rec_, defined commonly as the time required to reach within 10% of the final response. The heterojunction exhibits a τ_Res_ four times that of the Schottky contact and the τ_Rec_ is 5–8 times longer, i.e., the heterojunction device is slower. This is due to the traps in the transport mechanism, which is to be discussed subsequently in detail, and is a design compromise for the sensitivity enhancement.

## 4. Discussion

**Figure 6:**
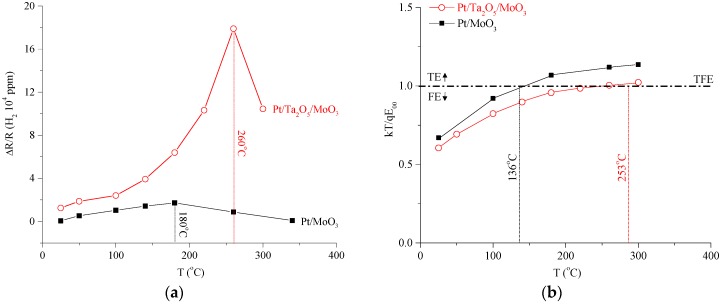
(**a**) Resistance change (ΔR/R) sensor response across temperature, as evaluated from I–V data of the Pt/MoO_3_ Schottky contact and the Pt/Ta_2_O_5_/MoO_3_ heterojunction. (**b**) Calculated kT/qE_00_ vs. T. kT/qE_00_ = 1 indicates Thermionic Field Emission (TFE) is the dominant transport mechanism, which correlates strongly with the largest resistance change for both devices, therefore providing a theoretical explanation for the optimal sensing temperature.

Sensitivity (S), commonly defined as the change in resistance over the base resistance, i.e., ΔR/R, is presented in Figure 6a, which is another way of viewing the I–V data of Figure 3. The maximum S for the Schottky contact is 1.92 at 180 °C, whereas that of the heterojunction is 17.96 at 260 °C. From Figure 6a, two noteworthy points can be extracted: (i) sensitivity is highly dependent on temperature, and (ii) the introduction of Ta_2_O_5_ enhances sensitivity six-fold and simultaneously shifts the optimal sensing temperature.

**Figure 7:**
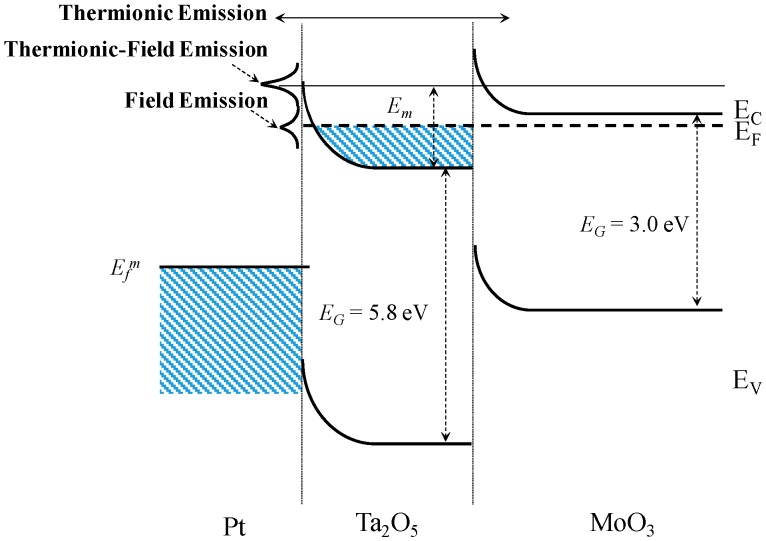
Band diagram of a metal-semiconductor Schottky heterojunction, with dominant transport mechanisms at various energy levels.

To explain the dependency of sensitivity on temperature, we focus the investigation on transport mechanism. The mechanism of gas sensing in terms of molecular interactions has been well studied, as in the comprehensive reviews [28,29,30,31]. Figure 7 depicts the band diagram of a metal-semiconductor Schottky barrier, with dominant transport mechanisms at various energy levels labeled. In the diagram, E_F_, E_C_, E_F_, and E_G_ denote the Fermi, conduction, valence, and bandgap energy levels, respectively. Transport is primarily influenced by two major factors: (i) presence of energy states within the bandgap (e.g., due to defects), and (ii) temperature. Under certain conditions, it may be possible for electrons with energies below the top of the barrier to penetrate the barrier by quantum-mechanical tunneling. This may modify the thermionic process in one of two ways. In the case of a very heavily doped or defected semiconductor at relatively low temperature, the current in the forward direction arises from the tunneling of electrons with energies close to the Fermi energy in the semiconductor, which is known as field emission (FE). If the temperature is raised, electrons are excited to higher energies and the tunneling probability increases rapidly because the electrons see a thinner barrier. On the other hand, the number of electrons carrying a certain energy level decreases rapidly with increasing energy. Therefore, there is a maximum contribution to the current from electrons having energy E_m_, which gives rise to thermionic field emission (TFE). If the temperature is raised further, virtually all of the electrons have enough energy to go over the top of the barrier, and then the effect of tunneling is negligible; therefore, pure thermionic emission (TE) is achieved. It is apparent that both temperature and the presence of intermediate energy states play a significant role in the type of transport in metal-semiconductor interfaces. 

With this work, TFE theory is, for the first time, utilized to analyze the transport of nanostructured oxide heterojunction. This is in contrast with the conventional attribution to either pure TE or pure FE [32,33]. The attribution of carrier transport to TFE for nanostructured interfaces is highly appropriate as the large amount of morphological edges create a large density of defects, which significantly aids transport when an appropriate amount of thermal energy and an applied electric field (via biasing) is available [34]. In TFE theory, the diffusion potential E_00_ describes the energy acquired by an electron for the thermally driven tunneling process to take place through a defect-rich interface, such as a nanostructured contact [35]. It is convenient to assess the extent of TFE by comparing the thermal excitation energy kT against the characteristic charge potential qE_00_. When kT/qE_00_ approaches unity, TFE is the dominant transport mechanism. Otherwise, when kT/qE_00_ is less than or greater than unity, the dominant transport mechanisms are FE and TE, respectively. Analytically, contact I–V characteristics and E_00_ can be related as follows [36,37]:

(1) $$I=I_{S}\;\exp\left(qV/E_{00}\right)$$

(2) $$I_{S}=A^{*}T^{2}\frac{{\left(q\pi E_{00}\right)}^{1/2}}{kT}\;{\left[V_{R}+\frac{\phi_{B}}{\cosh^{2}\left(E_{00}/kT\right)}\right]}^{1/2}\exp\left(\frac{-\left[q\phi_{B}\right]}{E_{00}}\right)\exp\left(\frac{qV_{R}}{E_{00}}\right)$$

(3) $$E_{00}=\frac{qV}{ln\left(I\right)-ln\left(I_{S}\right)}$$

where *I_s_* is the saturation current, *V_R_* is the reverse bias voltage, A* is the effective Richardson constant, *q* is the elementary charge, *φ*_B_ is the barrier height, *k* is Boltzmann’s constant, and *T* is the absolute temperature. The kT/qE_00_ ratio can be calculated by first calculating E_00_ using Equation (3), where *I_s_* is the y-intercept of the slope of the I–V curve taken at a voltage that the device is turn-on (in this case, 1 V).

From the perspective of transport, for both the fabricated Pt/MoO_3_ Schottky contacts and the Pt/Ta_2_O_5_/MoO_3_ heterojunctions, the value of kT/qE_00_ was calculated across a wide temperature range, presented in Figure 6b. It was found that, for the Pt/MoO_3_ contact, as kT/qE_00_ >> 1 for most of its operating temperature range, transport is mostly via FE, which is consistent with other MoO_3_ nanostructures. For the Pt/Ta_2_O_5_/MoO_3_ heterojunction, at low and high temperatures, transport is via FE and TE, respectively. At temperatures around 260 °C, as kT/qE_00_ ≈ 1, TFE becomes the dominant transport mechanism. Importantly, comparing Figure 6a,b, it is evident that the temperature at which sensitivity reaches the maximum correlates with the temperature at which TFE is dominant, which occurs for both structures. The correlation is surprisingly strong, i.e., 173 °C vs. 180 °C and 244 °C vs. 260 °C for the Schottky contact and heterojunction, respectively. Perhaps counterintuitively, these results provide strong indications that the defects introduced by nanostructure engineering, which is often viewed as a pure disadvantage, in fact contribute to the sensitivity enhancement.

## 5. Conclusions

In this work, through the characterization of Pt/MoO_3_ Schottky contacts and Pt/Ta_2_O_5_/MoO_3_ heterojunctions, we present previously unreported evidence of the connection between thermionic field emission and optimal gas sensing temperature. In fact, the kT/qE_00_ ratio can be regarded as a remarkably accurate predictor of the optimal sensing temperature. By attributing TFE as the main transport mechanism for nanostructured Schottky barrier gas sensors, this work provides an alternative explanation to the gas sensitivity improvement given rise by defect-rich nanostructured interfaces.
